# Primary Hepatic Neuroendocrine Tumor: What Do We Know Now?

**DOI:** 10.4021/wjon341w

**Published:** 2011-10-28

**Authors:** Benjamin Quartey

**Affiliations:** a National Capital Consortium, National Naval Medical Center, Department of Surgery, 8901 Wisconsin Ave, Bethesda, MD 20889, USA

**Keywords:** Primary hepatic tumor, Neuroendocrine tumor, Carcinoid, Liver

## Abstract

Primary hepatic neuroendocrine tumors (PHNETs) are rear neoplasm. Diagnosis is an evolution, and requires a systematic clinical exclusion with histological confirmation. Treatment is surgical with excellent prognosis, and a long-term follow-up is required due to high tumor recurring rate. Knowledge from this species of tumor remains limited due to paucity of cases. This article elaborates the key features, diagnosis algorithm, current management, other treatment options and extensive review of literature on this rear tumor.

## Introduction

Neuroendocrine (carcinoid) tumors (NETs) are rear heterogeneous group of neoplasm derive from neuroendocrine system first described by Oberndorfer in 1907; a German pathologist who coined the term *Karzinoide* to describe their carcinoma-like but benign and indolent nature [1]. Although NETs are distributed throughout the body, 54.5-75% are found in the gastrointestinal tract [2, 3] with 44.7%, 19.6%, 16.7%, 10.6% and 7.2% in the small intestines, rectum, appendix, colon and stomach respectively [3]. However, NETs makes about 1-2% of all gastrointestinal tumors [4] and the liver is the most common site of metastases [2, 3]. Primary hepatic neuroendocrine tumors (PHNETs) are a rarity and represents about 0.3% of all neuroendocrine tumors [5].

Due to the rarity of PHNETs, diagnosis is a continuum: *pre-operatively, intra-operatively, and post-operatively*. Despite advances in radiographic imaging, the current technology is still inadequate in making a definite diagnosis preoperatively. Intra-operative anatomical hepatic resection is therefore the recommended treatment of choice. Post surgical recurrence is an issue, but prognosis is excellent. This review highlights the current diagnosis criteria, management, other treatment options and review of literature on PHNETs.

124 reported cases of PHNETs (this include PHNEC and PHCT) were indentified in the literature [6-76]. The mean age at diagnosis was 51.9 years (standard deviation (SD) ±16.5), and no clear gender preference exist (50.8% female and 49.2% male). 73.3% of the cases were symptomatic and abdominal pain (65%) was the common complaint. Most tumors were single lesions (76.3%) with size ranging from 1.5-27 cm, located usually on the right lobe (48.4%) and diagnosed by histopathologic examination of the resected specimens (77.4%). 84.5% underwent surgery with mean disease free interval of 33.6-month and 5-year survival of 75%. 28.8% died after a mean follow up of 41 months. Most tumors stained positive for chromogranin A and endocrinologically silent.

## Origin and Classification

The origin of PHNETs continues to be an ongoing investigation and a mystery with three theories put forward for the past decade. These theories includes: (1) possible transformation of liver malignant stem cells [7]; (2) differentiation of ectopic heterotopic pancreatic or adrenal tissue located in the liver [8]; and (3) transformation of neuroendocrine cells in the epithelium of the intrahepatic biliary duct [78]. The biliary duct theory is the most favored and more accurate since the bile duct contain neuroendocrine argentaffin cells [9, 10], and the assumption that chronic inflammation in the biliary system can induced intestinal metaplasia leading to the development of neuroendocrine tumor [79].

NETs used to be classified based on tumor location as foregut, midgut, and hindgut NETs. This classification is complicated by the nomenclature “carcinoid” and “neuroendocrine tumors”. To ensure uniform communication, the World Health Organization (WHO) in 2000 adopted the term neuroendocrine tumors to describe both neuroendocrine and carcinoid tumors. The panel also adopted three classification of neuroendocrine tumors based on tumor morphology, vascular invasion and proliferative index. These sub-types includes: well-differentiated neuroendocrine carcinoma (no local invasion or metastases: typical carcinoid); moderately differentiated neuroendocrine carcinoma (atypia, local invasion or metastases: atypical carcinoid) and poorly differentiated neuroendocrine carcinoma (highly atypical cells, lymph node or distance metastases: small cell carcinoma) [80].

Therefore, all reported cases of primary hepatic carcinoid tumors (PHCT), primary hepatic neuroendocrine tumors (PHNETs) or primary hepatic neuroendocrine carcinoma (PHNEC) are all the same entity. Unfortunately, the term carcinoid and neuroendocrine carcinoma is still being used to describe low grade malignant tumor with minimal pleomorphism and malignant epithelial neoplasm with high mitotic activity, necrosis and invasion respectively.

## Incidence

Despite increase in the incidence of neuroendocrine tumor over time [2, 81], PHNETs remains a rarity, although possible [2] with less than 130 reported cases in English literature to the best of my knowledge [6-76]. This number includes both PHCT and PHNEC. However, most of the reported cases were PHCT in a form of retrospective case series and reports [6, 7, 9-13, 15-24, 26-49, 51-54, 60-68, 70-76]. The estimated incidence rate for carcinoid tumor in the United States was 6.25 cases per 100 000 people per year [2, 6].

## Clinical Features

PHNET usually does not present with any specific clinical features, but the common reported symptoms are due to mass effect of the liver and adjacent organ. These include vague abdominal pain, jaundice, palpable right upper quadrant mass, weight loss, and diarrhea. Abdominal pain is the most common complaint in symptomatic patients [6, 11, 81, 82] and 65% of the cases reviewed in this series reported this finding. Surprisingly, less than 20% of patients with PHNETs presents with classic carcinoid syndrome [11, 81, 82], described as skin flushing, abdominal pain and episodic diarrhea. 6.8% of the cases analyzed in this review had true carcinoid syndrome. Although it’s unclear why PHNETs are endocrinologically silent, others attribute this to spillage of hepatic enzymatic degradation of neoplastic-derived products directly into portal circulation [12].

Among the reported cases of PHNETs, female are affected slightly more often than men (50.8% versus 49.2%), with a mean age of 51.9 years (range 8-89 years), and the highest incidence was in fifth decade similar to previous report [13, 81, 82]. Most tumor were solitary (76.3%) but can be multicentric, and right lobar (48.4%) preference exist. Extrahepatic metastasis is about 18.6% and common sites include lung, bone and brain [13].

## Laboratory Data

Although PHNETs are hepatic in origin, standard serological marker such as apha-fetoprotein (AFP), carcinoembryonic antigen (CEA), and cancer-antigen (CA) 19-9 are non-diagnostic and not helpful [12]. Serum 5-hydroxytryptamine (5-HT) or 24-hour urine of 5-hydroxyindoleacetic acid (5-HIAA) may be effective but inferior to serum Chromogranin A in diagnosing NEC. Their respective sensitivity and specificity are (73% versus 87-100%) and (90% versus 92%) [12]. Chromogranin A is therefore considered the most useful marker to confirm neuroendocrine tumors including neuroendocrine carcinoma and carcinoids.

## Diagnosis

Since the first documented PHNET by Edmondson 53-years ago [17], diagnosis of this rear oncological entity is still a mystery. Diagnosis is a continuum starting from preoperative to post surgical including long-term follow-up in search of extra-hepatic primary.

Preoperative diagnosis of PHNETs with needle biopsy has been reported [8, 10, 11, 13, 18, 23, 28, 29, 36, 52, 62, 68, 72], but the diagnostic accuracy is not high enough. Needle biopsy was used in several cases and confirmed with surgical resection but this technique was accurate in making diagnosis in 11.3% (14/124) of the cases reviewed. Hwang et al reported 57.1% (4 out 7 patients) diagnostic accuracy in their small series, however the author caution against use of this diagnostic method [23]. The low diagnostic accuracy is evident in reported cases of misdiagnosis of PHNETs as hepatocellular carcinoma or cholangiocarcinoma [18-21, 24, 34, 44, 45]. Moreover, the literature is still unclear on the value of liver biopsy therefore post-surgical histological and immunohistochemical evaluation serves as the main method for the final diagnosis.

The objectives of preoperative evaluation is to actively exclude extrahepatic primary source since NET metastases to the liver are more common than PHNETs [2, 20, 21]. This approach is a stepwise, meticulous search for extrahepatic lesion beginning with ultrasound, computed tomography (CT) and magnetic resonance imaging (MRI) followed by octreoscan, upper and lower gastrointestinal endoscopy and diagnostic laparotomy [22]. Nevertheless, there is 16% failure rate to locate extrahepatic carcinoid despite extensive systematic investigation [85].

A traditional ultrasound will show multiple cystic lesions or solid mass [8, 20-23, 24, 26, 29, 31, 37, 52], and although not specific, it is helpful for differential diagnosis for hepatocellular carcinoma which appears as colliquation necrosis on ultrasound [24, 25, 27, 73]. CT scan will also reveal a cystic lesion with tumor enhancement due to the tumor’s hypervascularity [8, 20, 24-26, 29]. Utility of angiogram is questionable but can be helpful in elucidating the hypervascularity and central location of PHNETs [20, 21, 24, 26, 34, 44]. Hypointense and hyperintense T1 and T2-weighted images of MRI respectively are better in characterizing these lesions and currently being used more often [24-27]. However, PHNETs have a wide spectrum of appearance on MRI with benign and malignant features making preoperatively diagnosis still inaccurate [27].

PET-scanning with ^11^C-5 hydroxytryptophan tracer which concentrate highly in carcinoid tumors allowed for the identification of primary tumor in 84% (16 of 19 patients) of the cases reported by Orlefors et al [84]. The technology is however limited to few medical centers. Among the radiographs discussed above, octreoscan scintigraphy is the most efficient and ideal with specificity of 83% [25, 85], and can detect extrahepatic lesion and recurrence [25, 85, 86]. Donadon et al reported 88% specificity, 83% accuracy and 100% positive predictive value for octreoscan in detection of PHNETs in their series [15]. For complete work up, both upper and lower endoscopy is recommended [22].

Nevertheless, there is 16% failure rate using the above preoperative modalities to locate extrahepatic primary lesions [87], making surgical resection the most commonly used method and considered the treatment of choice [6, 10, 13, 15, 81, 82]. Sometimes, a very close post surgical long-term follow-up is required for definite diagnosis of primary hepatic lesion [15].

## Differential Diagnosis

The differential diagnosis is broad and the most common consideration include metastatic neuroendocrine tumor, hepatocellular carcinoma, small cell lymphocytic lymphoma, cholangiocarcinoma with neuroendocrine differentiation, metastatic Merkel cell carcinoma, and epithelioid variant of gastrointestinal stromal tumor [28].

## Pathology and Diagnosis

Among the reported cases, PHNETs ranges from 1.5-27 cm with a mean of 9.4 cm (SD ± 5.8) grossly, and they are generally gray-yellow in color and well demarcated lesion with multiple irregular hemorrhagic areas, but with cystic features ocassionally [8, 9, 29]. Microscopically, the tumor shows unique finding of insular, nested, trabecular or mixed pattern of cell growth pattern [6-76]. Immunohistological analysis is the most accurate diagnostic method due to high accuracy for detecting markers such as chromgranin A (CgA), neuro-specific enolase (NSE), neurilemmna cell S-100 protein and synaptophysin (SYP) [13, 28, 81, 82]. CgA was detected immunohistochemically in 94.7% of cases in this review comparable to the 84% reported by Iwao et al [13]. Therefore CgA is a sensitive index in diagnosing NEC [30, 80]. Some cases were analyzed histologically using Grimelius silver and Fontana-Masson stains [7, 9, 13, 19, 20, 24, 25, 28, 30, 31, 35-37, 45, 47]. These special stains can raise the diagnostic index although inferior to immunohistochemical analysis in diagnosing NECs.

## Surgical Management, Prognosis and Recurrence

The main treatment modality for PHNETs was surgical resection in 84.5% cases reviewed and the achieved 5-year survival rate was 75%. Knox et al reported 78%, 5-year survival rate in their 48 patient series [6] comparable to the 5-year, 74% survival rate reported by Iwo et al from the analysis of 53 patients with PHNETs [13]. The recurrence rate for both series was 18% comparable to the 19.8% reported in this series. Hwang et al linked the recurrence or prognosis of PHNETs to Ki67 (marker of tumor proliferation) index of < 2 % [18]. In their report, Ki67 was a prognostic factor for tumor recurrence, with a mean value in the non-recurrent group of 1.7% [18]. The longest disease-free time interval was 180 months [30] with a mean of 33.6 months.

Moreover, aggressive and major hepatic resection for HNETs is safe but the extent of the disease and type of surgery does not influence the survival rate [29]. The literature is limited with regards to extent of PHNETs burden and surgical resection and their influence on survival. Knox et al reported a comparable survival for patients with uni-lobar disease and bi-lobar disease, although the 10 year survivals were 88 % and 47 % respectively [6]. The extrahepatic involvement such as bone, lymph node and lung in their series was 60%, 60% and 40% respectively [6]. As shown in Table 1, the collective (bone, lung, lymph node) extrahepatic involvement was 18.6%. Metachronous lymph node metastasis is a major concerned with PHNETs but this can be managed with lymphadenectomy with good results [21]. Overall, surgical resection is effective, safe and prognosis is excellent despite high recurrence rate.

**Table 1:** Characteristics of 124 Cases of PHNETs

Characteristics	N* (%)
Age(years)	
Range	8-89
Mean	51.9 ± 16.5
Gender	
Male	61/124 (49.2%)
Female	63/124 (50.8%)
Presentation	
Symptomatic	88/120 (73.3%)
Abdominal Pain	57/88 (65.0%)
Abdominal Mass	11/88 (12.5%)
Jaundice	4/88 (45.0%)
Classic carcinoid syndrome	6/88 (6.8%)
Asymptomatic	32/120 (26.7%)
Method of diagnosis	
Surgery	94/124 (77.4%)
Biopsy	14/124 (11.3%)
Autopsy	14/124 (11.3%)
Tumor location	
Right lobe	60/124 (48.4%)
Left lobe	41/124 (33.1%)
Bi-lobar	23/124 (18.5%)
Tumor Size	
Range (cm)	1.5-27
Mean (cm)	9.4 (SD ± 5.8)
Tumor number	
Single	90/118 (76.3%)
Multiple	28/118 (23.7%)
Management	
Liver resection	98/116 (84.5%)
Liver transplant	3/116 (2.6%)
Chemotherapy	7/116 (6.0%)
Transcatherer arterial embolization(TACE)	5/116 (4.3%)
Radiofrequency ablation	1/116 (0.8%)
Immunohistochemical stains	
Chromogranin A	90/95 (94.7%)
Synaptophysin	48/95 (50.5%)
Cytokeratin	13/95 (13.7%)
Neuron specific enolase	46/95 (48.4%)
Disease free interval (months)	
Minimum	1
Maximum	180
Mean	33.6
Recurrence	
Recurrence	23/116 (19.8%)
No recurrence	93/116 (80.2%)
Survival	
Death	34/118 (28.8%)
Alive	84/118 (71.2%)
Follow up (months)	
Longest survival	> 192
Shortest survival	< 1
Mean survival	41
Metastases	
Metastase	21/113 (18.6%)
No metastases	92/113 (81.4%)
Survival	
1 year	82.0% (CI 0.74-0.89)
5 years	75.0% (CI 0.66-0.83)
10 years	73.4% (CI 0.64-0.82)

*This is based on detail of the individual cases reviewed.

## Patient Selection for Surgery

Eight years ago, Yao et al provided a standardized algorithm for selecting patient who will benefit from surgical resection and those proposal remains the current benchmark diagnostic tool. Their recommendation was based on four premises: primary or secondary hepatic lesion; extent of associated functional symptoms; intrahepatic and extrahepatic tumor burden, and information about tumor biology if possible [87]. The authors suggested an extensive, meticulous search for extrahepatic lesion before surgical resection is entertained, and if no other primary tumor is found at the time of liver resection, a close follow-up with imaging should continue since it may uncover an occult primary tumor within a few years. In addition, patient with functional symptoms due to tumor byproduct such as carcinoid syndrome should undergo surgical resection before other non-effective adjuvant therapies, and the main contraindication to surgery should be an anticipated incomplete resection except when tumor debulking was the initial surgical intent [87]. Finally, a good knowledge of the tumor biology in-question is very critical in patient selection since NECs are less aggressive with slow disease progression [87].

## Other Therapies

There are several adjuvant therapies being used in the management of PHNETs, however their role and effectiveness is still unknown. Andreola et al reported the case of a 19-year woman who was diagnosed with unresectable carcinoid tumor of the liver with metastases to regional lymph node and common hepatic duct [19]. Systemic chemotherapy with 5-fluorouracil lead to tumor down-staging and subsequent resection. In the same series, two other patients failed to respond to systemic chemotherapy [19]. Approximately 6% of the cases reviewed were treated with chemotherapy as the main treatment modality [9, 10, 19, 32, 33, 59]. The main use of chemotherapy is for unresectable lesions and tumors with distant metastases [7-9, 18, 19, 34, 56, 58]. So far the paucity of cases makes it difficult to also evaluate the effectiveness of transarterial chemo-embolization (TACE). This modality has been used in downstaging unresectable lesion [40, 45-47, 66, 67], and recurrent tumor post resection [13, 19, 21, 25].

Moreover, the effectiveness of radiofrequency ablation and percutaneous ethanol injection treatment (PEIT) are limited. Huang et al reported the application of PEIT in a patient with three recurring tumors 30 days after resection with good response and disease free interval of 13 months after treatment [25]. Finally, no clear indication for liver transplantation exists, but transplantation has been performed in few patients with unresectable lesion, multiple liver lesion, and large tumor burden with success [22, 76]. At the moment, surgical resection seems to be the only effective management available and the treatment of choice [6, 15, 18, 25, 88].

## Conclusion

Despite the liver being the common site for metastases from other gastrointestinal NEC or other tumors, primary hepatic neuroendocrine tumors are rear but can occur. PHNETs are slightly common in female, with mean age of 51.9 years, and presents with vague abdominal pain. The tumor has right lobar preference and endocrinologically silent. Diagnosis entails a systematic and painstakingly searches for extrahepatic source using preoperative modalities such ultrasound, CT scan, MRI, PET scan, octreoscan, and endoscopy. Inability to make any preoperative diagnosis or find any extrahepatic lesion is a common scenario and sometimes the only method of diagnosis is effective, close follow-up with imaging after surgical resection. In most cases tumor cells are immunohistochemically positive for CgA. Surgical resection is the treatment of choice and more prospective cases are needed to assess other therapies.
